# Roadmap for systematic identification and analysis of multiple biases in causal inference

**DOI:** 10.1093/aje/kwag166

**Published:** 2026-07-16

**Authors:** Rushani Wijesuriya, Rachael Hughes, John B Carlin, Rachel L Peters, Jennifer J Koplin, Margarita Moreno-Betancur

**Affiliations:** Clinical Epidemiology & Biostatistics Unit (CEBU), Murdoch Children's Research Institute, Parkville, Victoria, Australia; Clinical Epidemiology & Biostatistics Unit (CEBU), Department of Paediatrics, University of Melbourne, Parkville, Victoria, Australia; MRC Integrative Epidemiology Unit, University of Bristol, Bristol, United Kingdom; Population Health Sciences, Bristol Medical School, University of Bristol, Bristol, United Kingdom; Clinical Epidemiology & Biostatistics Unit (CEBU), Murdoch Children's Research Institute, Parkville, Victoria, Australia; Clinical Epidemiology & Biostatistics Unit (CEBU), Department of Paediatrics, University of Melbourne, Parkville, Victoria, Australia; Department of Paediatrics, University of Melbourne, Parkville, Victoria, Australia; Population Health, Murdoch Children’s Research Institute, Parkville, Victoria, Australia; Population Health, Murdoch Children’s Research Institute, Parkville, Victoria, Australia; Child Health Research Centre, University of Queensland, Brisbane, Queensland, Australia; Clinical Epidemiology & Biostatistics Unit (CEBU), Murdoch Children's Research Institute, Parkville, Victoria, Australia; Clinical Epidemiology & Biostatistics Unit (CEBU), Department of Paediatrics, University of Melbourne, Parkville, Victoria, Australia

**Keywords:** causal inference, ideal trial, quantitative bias analysis, multiple bias modeling, simultaneous bias analysis, selection bias, confounding bias, measurement bias

## Abstract

Observational studies examining causal effects rely on unverifiable assumptions, the violation of which can induce multiple biases. Quantitative bias analysis (QBA) methods examine the sensitivity of findings to such violations, generally, by producing estimates under alternative assumptions, incorporating additional information. Although substantial guidance exists for implementing QBA, there is limited guidance on how to systematically determine the assumptions underlying a primary causal analysis and the potential violations that should guide bias analysis. Consequently, many assumptions remain implicit, leading to selective and therefore misleading QBA. To address this gap, we propose a roadmap for systematically identifying and analyzing multiple biases. Briefly, this consists of (1) articulating the assumptions underlying the primary analysis through specification of the “ideal trial” that defines the causal estimand and the assumptions required by its (possibly implicit) emulation, depicting these assumptions using a causal diagram; (2) extending the diagram to depict alternative assumptions, under which biases may arise; (3) obtaining a single estimate that simultaneously corrects for all potential biases. We illustrate the roadmap using an investigation of the effect of breastfeeding on the risk of childhood asthma and, through simulations, demonstrate the need for analyzing multiple biases jointly rather than one at a time.

## Introduction

Many epidemiological studies aim to quantify the causal effect of an exposure on an outcome, generally relying on causal assumptions such as exchangeability, consistency, and positivity. Violations of causal assumptions can lead to systematic causal bias—a discrepancy between the expected value of the causal effect estimate and the true (unknown) value—versions of which are often categorized as confounding, measurement (or information), and selection bias.^1^ For example, confounding and selection bias may arise as a violation of exchangeability assumptions.^2^

Quantitative bias analysis (QBA) examines the sensitivity of findings to violations of the assumptions made in the primary analysis, generally by producing estimates under alternative assumptions.^1^ Specifically, QBA focuses on alternative assumptions under which the observed data alone do not permit unbiased estimation of the causal effect. Therefore, QBA relies on analytic approaches that incorporate additional information to address these biases.

Although substantial guidance has emerged for conducting QBA, including analytic approaches and best practice recommendations,^1^^,^^3-5^ there is currently limited guidance on how to design a bias analysis by systematically identifying the assumptions underlying a primary causal analysis and the potential violations of those assumptions that are likely sources of systematic bias. Given the lack of a structured approach, in many cases the primary analysis assumptions remain implicit, which results in selective identification and consideration of biases in QBA, undermining the transparency and completeness of bias assessments.

In addition, even when multiple potential biases are identified, a common strategy for QBA in practice involves analyzing each one at a time (producing multiple alternative estimates).^1^^,^^3^ Given that different bias sources are not necessarily unrelated or result in bias in the same direction, this strategy can lead to misleading conclusions about the overall impact of biases.^1^^,^^5^ Alternatively, multiple bias modeling approaches can be used, which account for interrelationships between different sources of bias. However, this can be a complex undertaking as it requires simultaneously specifying model(s) for multiple biases and their parameters.^3^ To set up appropriate bias models, it is essential to understand the overall causal structure of the problem.

In this work, we propose a roadmap for systematically identifying and analyzing multiple biases (see Box 1) and illustrate the roadmap using a case study investigating the effect of breastfeeding on the risk of childhood asthma. Further, using simulations based on this case study, where multiple dependent biases are plausible, we compare the commonly used “one-at-a-time” bias analysis with simultaneous bias analysis (a type of multiple bias modeling) to illustrate the importance of analyzing multiple biases jointly and to motivate the use of the proposed roadmap.

Box 1
**Roadmap for quantitative bias analysis considering multiple biases simultaneously.**

- **Step 1**: Articulate all identifiability assumptions made in the primary analysis by specifying the ideal trial (i.e., the trial that defines the causal effect of interest) and considering the assumptions required to emulate it, depicting these assumptions using directed acyclic graphs (DAGs) where possible.
- **Step 2:** Consider plausible violations of the assumptions in the primary analysis due to variables that are unobserved/incomplete/mismeasured in the study (e.g., unmeasured confounding, gold standard measures, etc.) and depict these alternative scenarios using DAGs to assess possible sources of causal biases in the primary analysis.
- **Step 3**: Obtain a single estimate assessing uncertainty to all possible sources of bias identified by applying a simultaneous multiple bias analysis approach.

## Preliminaries: illustrative case study example and target estimand

We consider a case study,^6^ from the HealthNuts cohort study, a prospective observational study of 5279 infants aged 11-15 months in Melbourne, Australia from 2007 to 2011 and followed up at ages 4, 6, and 10 years.^7^ We focus on the question: what is the impact of receiving any (vs no) breastfeeding (direct or expressed with any duration or frequency) in the first 12 months on the risk of asthma at 6 years? This question is of interest because some organizations recommend that any breastfeeding is better than none,^8-10^ where “breastfeeding” is generally interpreted as feeding breast milk by any mode (i.e., including expressed breast milk).^8^^,^^11^

Let *A* denote any breastfeeding in the first 12 months (*A=1*) or not (*A=0*), and *Y* denote the binary outcome, with *Y=1* if child is diagnosed with asthma by age 6, and *Y=0* otherwise. Although *A* summarizes a time-varying behavior over 12 months, under the plausible assumption that breastfeeding is either initiated in the first 1 to 2 weeks or is never initiated,^12^^,^^13^ “any breastfeeding” can be treated as a (single time-) point exposure, which is the focus of this paper.

Our target estimand is the average causal effect (ACE) of any breastfeeding in the population of all neonates born in Melbourne who may be able to be breastfed, specifically the causal risk ratio $\mathrm{P}\left({Y}^1=1\right)/\Pr \left({Y}^0=1\right)$, where ${Y}^a$ is the potential (a.k.a. counterfactual) outcome when intervening to set the exposure *A* to the value *a*.

We note that there is a threat to the consistency assumption in this case study arising from the fact that the multiple versions “any breastfeeding” (e.g., duration, mode) may not be irrelevant to the outcome. In such settings, where it can be assumed that treatment precedes version, it has been shown^14^ that the target estimand (overall treatment effect in the target population, with its distribution of versions) is identifiable using the ordinary identifiability formula under stricter positivity and conditional exchangeability assumptions than in settings with no multiple versions of treatment. Specifically, positivity and exchangeability need to consider a set of confounders $\boldsymbol{C}$ that includes variables, which are common causes of treatment and version of treatment in addition to common causes of treatment and outcome. Under these assumptions, and in the absence of missing data and measurement error, the causal risk ratio is identifiable from observable data based on the usual g-formula: $P\left({Y}^a=1\right)={\sum}_{\boldsymbol{c}}P\left(Y=1|A=a,\boldsymbol{C}=\boldsymbol{c}\right)\times P\left(\boldsymbol{C}=\boldsymbol{c}\right)$**.**^15^

This implies that unbiased estimation is possible using a number of methods,^15^ e.g., outcome regression,^16^ with mean specification as follows:


(1) \begin{equation*} \log \left[\Pr \left(Y=1|A=a,\boldsymbol{C}=\boldsymbol{c}\right)\right]={\beta}_0+{\beta}_1A+{\boldsymbol{\beta}}_{\mathbf{2}}^{\prime}\boldsymbol{C}, \end{equation*}

where ${e}^{\beta_1}$ equal the causal risk ratio, if the outcome model in eqn (1) is correctly specified and there is no effect measure modification across strata of $\boldsymbol{C}$ (in addition to the above-mentioned assumptions)**.**

## The proposed roadmap for multiple bias analysis

### Step 1: Systematic determination of all assumptions in the primary analysis and depicting these using directed acyclic graphs

To facilitate this step, we propose defining the ideal randomized trial that defines the causal effect of ultimate interest. Specifying the ideal trial is an alternative but equivalent way to the potential outcomes framework for precisely specifying the relevant estimand.^17^

#### Using the ideal trial to articulate assumptions for the primary analysis

The ideal trial is a hypothetical trial with an infinite and representative sample, randomization, perfect adherence and follow-up, and no missing data or measurement error.^17^ It is defined by specifying its protocol components: eligibility criteria, treatment strategies, assignment procedures, follow-up period, and outcome measure.^18^ The observational analysis can then be seen as an attempt to emulate the ideal trial. The emulation of each component will rarely be perfect, with systematic bias avoided only under unverifiable identifiability assumptions. By considering the emulation of each protocol component in turn, we can determine all the identifiability assumptions and assess their plausibility.

#### Directed acyclic graphs

Once the assumptions are systematically recognized, it is helpful to depict these using a directed acyclic graph (DAG) (where possible, as not all assumptions can be depicted in DAGs).^19^ Comprehensive reviews and tutorials on DAGs can be found elsewhere.^15^^,^^20^ The primary analysis of a study typically assumes that under the assumptions depicted in the corresponding DAG (and any additional assumptions that are not amenable to depiction in a DAG), standard (non-QBA) analytic approaches (e.g., eqn (1)) suffice to obtain unbiased estimates from the observable data.

#### Application to the case study

We begin by detailing features of the published analysis relevant for examining potential biases, along with notation for related variables. Firstly, the HealthNuts cohort included only children whose guardians consented (denoted by *S;* 1: yes, 0: no) and could speak and understand English (denoted by *E*; 1: yes, 0: no). Second, the study relied on parental reports of breastfeeding (${A}^{\ast }$), and asthma (${Y}^{\ast }$), as more objective measures (e.g., doctor diagnosis) were not collected. Third, a potential confounder, pre-exposure hypertension (*U*; 1: presence, 0: absence), was unmeasured.^21^ Fourth, substantial loss to follow-up, leading to missing data in ${Y}^{\ast }$ (indicated by ${R}_y$; ${R}_y=1$ if ${Y}^{\ast }$ observed and ${R}_y=0$ otherwise).

Table 1 outlines the protocol components for the ideal trial capturing the estimand of interest (column 1), as well as their emulation with HealthNuts data as implicit in the published primary analysis (column 2) and the corresponding identifiability assumptions (column 3).

**Table 1:** Ideal trial specification for systematically identifying potential causal biases in the breastfeeding and asthma case study.

**Protocol component**	**Ideal trial**	**Emulation (implicit in published analysis** ^6^)	Identifiability assumptions
Eligibility criteria	**Study sample** All neonates born in Melbourne who may be able to be breastfed	**Analytic sample selection**: All HealthNuts participants, who were infants aged 11-15 months in Melbourne in 2007-2011, recruited during routine 12-month immunization sessions across Melbourne, with parents/guardians who consented to participate in the study and could read and understand English. Inclusion was regardless of missingness in outcome, which was handled via inverse probability weighting (IPW) with weights estimated conditional on the selected confounders and measured exposure.^a^ Given lack of data, no exclusions made on the basis of infant not being able to be breastfed either due to (1) infant being unable to ingest or digest breastmilk or (2) mother unable to breastfeed.	The HealthNuts study participants are representative of (i.e., not systematically different from) the population of all infants born in Melbourne in 2006-2010 who may be able to be breastfed. Missingness in the outcome is independent of outcome given the measured exposure and the selected confounders. There are no HealthNuts participants who may not be able to be breastfed due to condition (1) detailed in the previous column of the table, and the causal effect of interest is not different (i.e., no effect modification) for infants who may not be able to be breastfed due to condition (2).
Treatment strategies	**Treatment strategies**: (1) Never breastfeed in the first 12 months (2) Breastfeed (direct or expressed, any duration or frequency) in the first 12 months	**Treatment strategies**: (1) Never breastfeed in the first 12 months (2) Breastfeed (direct or expressed, any duration or frequency) in the first 12 months Exposure/adherence measure: parent report of ever breastfeeding in the first 12 months collected when infant aged 11-15 months.	Parents can accurately recall and report breastfeeding directly or via bottle during the first year of life at 11-15 months.
Assignment procedures	**Classification of participants into treatment strategies**: Randomization at recruitment without blind assignment.	**Classification of participants into treatment strategies**: No randomization, no blinding. Participants classified into treatment arm consistent with parent report of ever breastfeeding in the first 12 months collected when infant aged 11-15 months.	The following set of variables (or more specifically, their measures) represents a sufficient set of confounders (including those that affect both treatment and version of treatment) to eliminate all confounding: child’s sex measured at birth, maternal age at birth, maternal smoking during pregnancy, parental ethnicity, SES, maternal asthma, paternal asthma, family history of allergy, number of siblings.
Follow-up period	Start: birth End: child aged 6 years	Start: child aged 11-15 months End: child aged 6 years	Time zero of follow-up and the timing at which eligibility was assessed are later than in the ideal trial and misaligned with the start of treatment (birth). This could lead to (1) misclassification bias and/or (2) selection bias^22^ (1) Misclassification bias: we assume that breastfeeding is either established in the first weeks or is never established, so the treatment strategies in the ideal trial are equivalent to “initiate breastfeeding” vs “do not initiate breastfeeding”. Thus, under this assumption, the treatment strategies in the ideal trial are clearly distinguishable at time zero, and as described in the text they can be treated as time-invariant (i.e., as point treatments). Additionally, this means that setting recall error apart (noted above) an indicator of ever breastfed in the first 12 months in the observational study can validly emulate the treatment assignment indicator in the ideal trial, thereby avoiding potential misclassification bias. (2) Selection bias: this is avoided under the assumptions already noted under “Eligibility criteria” (i.e., the HealthNuts participants recruited at age 11-15 months are representative of all infants born in Melbourne who are able to be breastfed).
Outcome	**Outcome measure**: Clinically diagnosed asthma at age 6 (i.e., physician assessment of clinical history, with assessment at age 6, in conjunction with lung function testing demonstrating expiratory airflow limitation)	**Outcome measure**: Parental report of doctor-diagnosed asthma and either wheezing episodes in the last 12 months or use of common asthma medication (relievers and preventers) in the previous 12 months when child aged 6 years.	Parent self-reports are accurate measures of clinically diagnosed asthma at age 6.

Abbreviation: SES, socioeconomic status

a Although the published study had missing data in exposure and confounders, here we assume them to be fully observed for brevity, as only a small proportion of data were missing (exposure: 2.4%; confounders: 13.2%). The resulting study sample size was *n* = 4579.

Figure 1 below shows the assumed (simplified) DAG under the primary analysis. See published investigation for assumed relationships between all measured confounders.^6^

**Figure 1:**
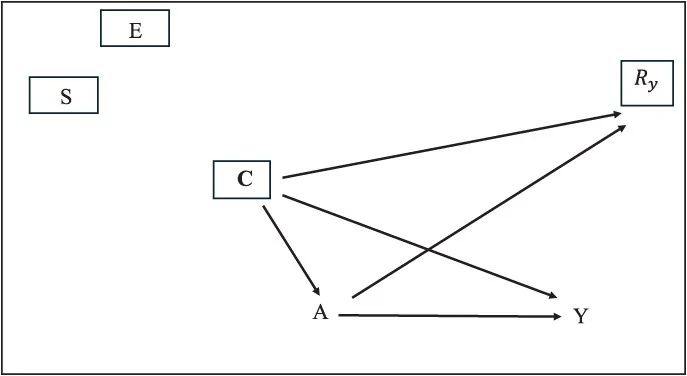
Directed acyclic graph (DAG) depicting the assumed causal structure in the published analysis.^6^  $\boldsymbol{C}$ includes child’s sex assigned at birth, mode of delivery, gestational age at delivery, low birth weight, maternal age at birth, maternal smoking during pregnancy, parental country of birth, family socioeconomic status, maternal asthma, paternal asthma, family history of allergic disease, and number of siblings. Of note, the boxes around variables indicate conditioning, and implicitly, the study assumed no measurement error (i.e., ${A}^{\ast }=A$ and ${Y}^{\ast }=Y$) and no effect modification by parents’ ability to speak and understand English (E) or consent (S). The response indicator ${R}_y$ is defined as ${R}_y=1$ if ${Y}^{\ast }$observed and ${R}_y=0$ otherwise.

### Step 2: Considering plausible violations of assumptions of the primary analysis and using DAGs to identify potential sources of bias

The second step of the roadmap involves considering plausible violations of the assumptions in the primary analysis and depicting these using DAGs.

#### Causal biases and their graphical structures

Causal biases are broadly categorized into 3 types: selection, confounding, and measurement bias (see Table 2). It is helpful to depict the different biases using DAGs. Doing so, may clarify how biases, particularly confounding and selection bias, enter an analysis and can help inform multiple bias model specification in Step 3 of the roadmap. See Figure 2 for a few example DAGs for different types of bias, although different DAGs are possible.

**Table 2:** Description and graphical structure of key causal biases.

Causal bias	Description	Graphical structure
Selection bias	Occurs when the sample used for analysis (i.e., analytic sample) is not representative of the population for which inference is of interest (i.e., target population) This can occur either because (1) the population included in the study (i.e., study sample) is not representative of the target population or, (2) because the analytic sample (the observed portion of the study sample) is not representative of the study sample, or (3) both.^13^	Broadly can arise in 2 ways **Collider stratification bias/Type I selection bias** Formally, in a DAG, the structure of this bias can be represented as arising from a non-causal (biasing) path that becomes open after restricting the analysis or conditioning on one (or more) level(s) of a common effect of 2 variables (known as a “collider”), one of which is either the exposure or a cause of the exposure, and the other is either the outcome or a cause of the outcome.^23^^,^^24^ In Figure 2(i) conditioning on ${R}_y$ opens a biasing pathway between *A* and *Y* via ${R}_y$. **Generalizability bias/Type II selection bias** This type of bias arises due to restricting to one (or more) level(s) of an effect measure modifier (a variable such that the magnitude of the causal effect differs across its strata). The causal structure of effect modification cannot be denoted as a biasing pathway but may be depicted as shown in Figure 2(ii) where *E* is an effect measure modifier of the causal effect of *A*on *Y* for illustration. Other depictions are also possible as illustrated elsewhere.^25^^,^^26^ This type of bias is scale dependent (multiplicative vs additive) as it arises due to effect measure modification.^25^^,^^27^
Confounding bias	Occurs due to uncontrolled common causes of the exposure and outcome	Formally, in a DAG, the structure of this bias can be depicted as resulting from an open non-causal (biasing) “backdoor” path between the exposure and the outcome. Figure 2(iii) shows an example of confounding bias, where there is an open backdoor path between *A* and *Y* via *U*
Measurement bias	Occurs due to discrepancy between the measured value of a quantity and its true value as determined by what is considered to be the gold standard instrument. This includes misclassification in the case of categorical data.	The structure of this bias can be denoted in a DAG by depicting both the measured variable and the underlying true variable (e.g., *A* and ${A}^{\ast}$). The specific structure of the measurement error varies depending on whether the errors are assumed to be differential or non-differential and/or dependent or independent (as defined below) and cannot always be represented as a biasing pathway.^28^ And there may be measurement bias even in the context of non-differential, independent measurement error. Measurement errors are said to be independent if the measured outcome and the exposure are statistically independent of one another conditional on the true outcome and the true exposure (i.e., ${Y}^{\ast}\perp{A}^{\ast}\mid Y,A$) and dependent otherwise. Measurement errors are said to be differential if the measured outcome (or measured exposure) depends not only on its own true value, but also on the true exposure (or true outcome), either directly or through the measured-true relationship varying with it, and non-differential otherwise. Specifically, 2 forms of differential error can be distinguised^29^: (i) additive form, where *A* (or *Y*) leads to a systematic shift in the ${Y}^{\ast }$ (or ${A}^{\ast }$), and (ii) multiplicative form, where the *A* (or *Y*) modifies the strength of the relationship between $Y-{Y}^{\ast }$ (or $A-{A}^{\ast}$). As such (2) cannot be depicted in a DAG. Figure 2(iv) provides an example of non-differential and dependent (via *C*) measurement errors.

Abbreviations: DAG, directed acyclic graph.

**Figure 2:**
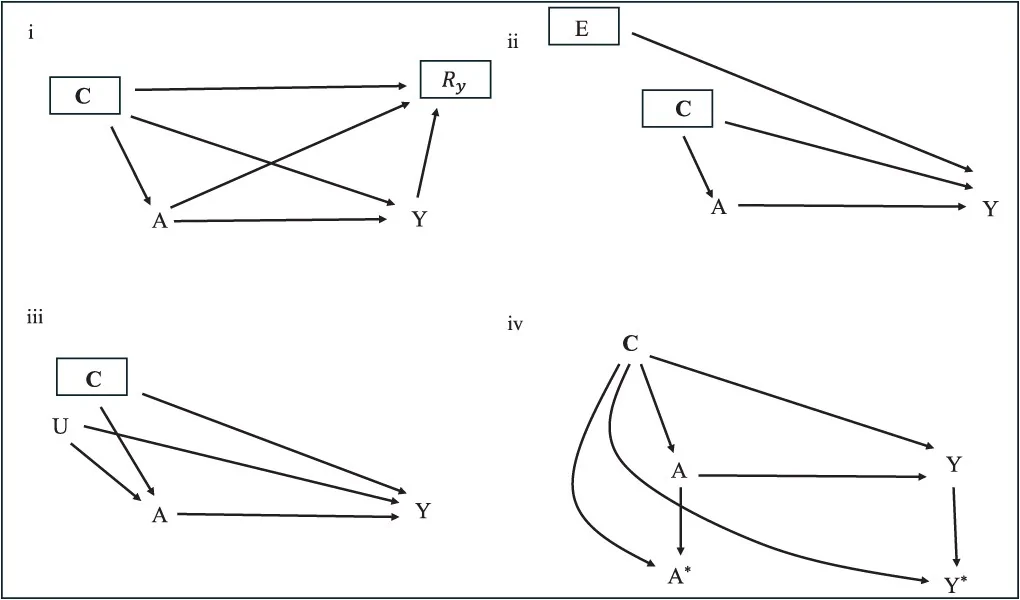
Directed acyclic graphs (DAGs) depicting examples of (i) collider-stratification bias due to conditioning on the response indicator ${R}_y$, (ii) generalizability bias due to restricting the analysis to one (or more) levels of the effect modifier *E *, (iii) confounding bias due to unmeasured confounder *U *, (iv) measurement bias due to differential and dependent measurement error in both ${A}^{\ast }$and ${Y}^{\ast }$ (if $\boldsymbol{C}$ not conditioned upon, as depicted). Note: A box around a node indicates conditioning/restriction to levels of the variable.

#### Application to the case study

We focus on the most plausible violations that arise due to variables, which are unobserved, incomplete, and/or mismeasured in the study (including unobserved data for those not selected into the study sample). This is followed by their structure in an alternative DAG (Figure 3), where alternative assumptions are shown in gray. Less plausible violations, along with justifications, are provided in Table S1.

**Figure 3:**
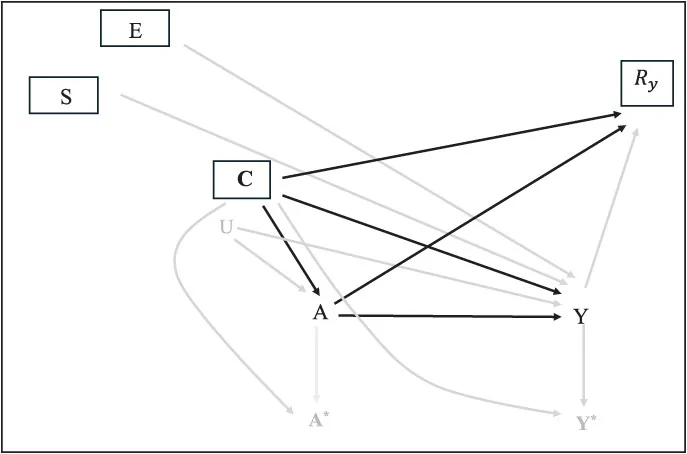
Directed acyclic graph (DAG) depicting a plausible alternative causal structure (all arrows) for the case study, which represents violations of assumptions made in the primary analysis (denoted using solid black arrows and assuming ${A}^{\ast }=A$ and ${Y}^{\ast }=Y$—see Figure 1).

##### Selection bias

The study was restricted to infants with guardians who could speak/understand English and consented *(E=1,S=1)*, but the primary analysis assumed no effect measure modification by these variables (Figure 1). However, parents who consented were more likely to have infants with a history of eczema, which is a potential effect measure modifier (i.e., the effect of breastfeeding on asthma could be different among those with eczema and no eczema).^6^^,^^7^ This implies a likelihood of effect measure modification by proxy for *S*, in which case the primary analysis estimate is subject to Type II selection bias (Table 2). Additionally, *E* (which is a strong proxy for ethnicity) is also a likely effect measure modifier by proxy, in which case a similar bias can arise. Figure 3 thus depicts the plausibility of *E* and *S* being effect measure modifiers in the risk ratio scale.

The primary analysis assumed that missingness in the outcome was only dependent on the observed variables, $\boldsymbol{C}$ and ${A}^{\ast }$. Figure 3 considers potential collider-stratification (selection) bias due to missingness in the outcome being additionally dependent on the outcome, which is plausible through unmeasured healthcare-seeking behaviors heightened by illness.

##### Confounding bias

The primary analysis adjusted for a set of measured confounders. Figure 3 depicts a potential residual confounding due to unmeasured gestational hypertension (*U *).^21^ Note that there may be other potential unmeasured confounders (including treatment-version confounders) that could be explored, but we focus on gestational hypertension for illustration.

##### Measurement error

The primary analysis assumes perfect measurement of exposure and outcome. Figure 3 depicts potential measurement error due to parent reports of the outcome and exposure. Although adjusting for $\boldsymbol{C}$ in the primary analysis excludes the possibility of bias due differential measurement error of an additive form (as defined in Table 2) and dependent measurement error, this does not entirely preclude measurement bias.^28^ In general, measurement error will lead to bias, except when the errors are non-differential and independent, and there is no causal effect of *A* on *Y*.^28^^,^^30^

### Step 3: Conducting quantitative multiple bias analysis

When multiple potential sources of bias are present, they are often analyzed one at time, but this can result in misleading results because it implicitly assumes the sources of bias act in isolation. Instead, a multiple bias modeling approach^1^ should be used.

One multiple bias modeling strategy is to analyze biases serially (often using single bias modeling approaches^1^) in the reverse order in which the biases are assumed to occur.^1^ For example, consider 2 biases for brevity: unmeasured confounding and mismeasured exposure. One might assume that unmeasured confounding occurs first, followed by exposure misclassification. Then, the true exposure is generated first based on observed data, followed by unmeasured confounder based on observed data and true exposure. In this way, dependencies among variables, as encoded in a DAG, are incorporated. However, because each step builds on previous corrections, the final estimate can vary depending on the order chosen,^1^^,^^4^^,^^31^ which is problematic when a plausible order is unclear. A more flexible strategy analyses the biases simultaneously without an a priori assumption about their order.^32^^,^^33^ Here, we adopt the simultaneous strategy, using a recent approach described in the next section.^33^ Simulations have shown that this approach performs similarly in terms of bias estimation to serial analysis when the latter is conducted using the “true” order of biases, under relatively simple scenarios.^32^^,^^33^

Regardless of the strategy used, all QBA approaches require bias parameters^1^^,^^34^ that stipulate the strength and direction of the association between observed and unobserved/mismeasured/incomplete variables under alternative assumptions. Different bias parameters are required for the different types of bias (e.g., see Table S2). By their nature, bias parameters are not estimable from observed data and must be informed by external sources (e.g., expert opinion, substantive literature, and external validation data) or internal validation data.^3^ Investigators may treat bias parameters in different ways (e.g., as deterministic or as arising from a distribution/probabilistic) and there are excellent texts providing further details,^1^^,^^3^ so we omit them here for brevity. Within a multiple bias analysis, a probabilistic treatment may be necessary for ease of presentation.^3^ Under this approach, referred to as probabilistic bias analysis (PBA), a (joint) prior probability distribution is assigned to the bias parameter(s) followed by a Bayesian analysis or utilization of a Monte Carlo sampling procedure.^1^^,^^3^ PBA allows incorporating the investigators’ uncertainty about the bias parameters in the final estimate, provided that the uncertainty is properly captured by the distribution used.

#### Simultaneous multiple bias modeling approach

The approach considered works by expressing the desired joint probability (or model), which involves unobserved/mismeasured/incomplete variables, as the product of the joint probability of the observed variables and a “bias-adjusting” probability that depends on bias parameters according to the assumed alternative assumptions. This enables derivation of an expression for the bias-adjusting probability that is then used to generate values for the full (ideal) data under the alternative assumptions and thereby obtain estimates under alternative assumptions as outlined below.^33^

1) **Derive an expression for the bias-adjusting probability and posit bias models**

In our setting, the desired joint probability is $\mathrm{P}\big(Y=y,A=a,\boldsymbol{C}=\boldsymbol{c},\ U=u\big)$ and the observed joint probability is $\mathrm{P}\big({Y}^{\ast }={y}^{\ast },{A}^{\ast }={a}^{\ast }, C=c\big|S=1,E=1,{R}_y=1\big)$. Let *π* denote the bias-adjusting probability such that the desired joint probability is the product of *π* and the observed joint probability. That is:


(2) \begin{align*} \pi =\frac{\mathrm{P}\left(Y=y,A=a,\boldsymbol{C}=\boldsymbol{c},U=u\right)}{\mathrm{P}\left({Y}^{\ast }={y}^{\ast },{A}^{\ast }={a}^{\ast },C=c\right|S=1,E=1,{R}_y=1\Big)\ }. \end{align*}

Based on the assumptions in Figure 3, *π* can be expressed as follows (see Appendix S1 for detailed steps):


(3) \begin{equation*} \pi =\frac{\mathrm{P}\left(U=u|A,Y\right)\times \mathrm{P}\left(Y=y|A,{Y}^{\ast },\boldsymbol{C}\right)\times \mathrm{P}\left(A=a|{A}^{\ast },{Y}^{\ast },\boldsymbol{C}\right)}{\mathrm{P}\left(S=1|{A}^{\ast },{Y}^{\ast}\right)\times \mathrm{P}\left(E=1|{A}^{\ast },{Y}^{\ast}\right)\times \mathrm{P}\left({R}_y=1|{A}^{\ast },{Y}^{\ast },\boldsymbol{C}\right)}. \end{equation*}

Parametric models are then posited for each of the factors in this expression, referred to as the bias models. With binary variables, logistic regression models can be used as follows:


$$ \mathrm{logit}\left(\mathrm{P}\left(A=1|{A}^{\ast },{Y}^{\ast },\boldsymbol{C}\right)\right)={\gamma}_0+{\gamma}_1{A}^{\ast }+{\gamma}_2{Y}^{\ast }+{\boldsymbol{\gamma}}_{\mathbf{3}}^{\boldsymbol{T}}\boldsymbol{C} $$

$$ \mathrm{logit}\left(\mathrm{P}\left(Y=1|{Y}^{\ast },A,\boldsymbol{C}\right)\right)={\alpha}_0+{\alpha}_1{Y}^{\ast }+{\alpha}_2A+{\boldsymbol{\alpha}}_{\mathbf{3}}^{\boldsymbol{T}}C $$

$$ \mathrm{logit}\left(\mathrm{P}\left(U=1|A,Y\right)\right)={\delta}_0+{\delta}_1A+{\delta}_2Y $$

$$ \mathrm{logit}\left(\mathrm{P}\left(S=1|{A}^{\ast },{Y}^{\ast}\right)\right)={\theta}_0+{\theta}_1{A}^{\ast }+{\theta}_2{Y}^{\ast }+{\theta}_3{A}^{\ast }{Y}^{\ast } $$

$$ \mathrm{logit}\left(\mathrm{P}\left(E=1|{A}^{\ast },{Y}^{\ast}\right)\right)={\lambda}_0+{\lambda}_1{A}^{\ast }+{\lambda}_2{Y}^{\ast }+{\lambda}_3{A}^{\ast }{Y}^{\ast } $$

(4) \begin{equation*} \mathrm{logit}\left(\mathrm{P}\left({R}_y=1|{A}^{\ast },{Y}^{\ast },\boldsymbol{C}\right)\right)={\eta}_0+{\eta}_1{A}^{\ast }+{\eta}_2{Y}^{\ast }+{\boldsymbol{\eta}}_{\mathbf{3}}^{\boldsymbol{T}}\boldsymbol{C} \end{equation*}

In the above model(s) for *S* (and *E*), an interaction between ${A}^{\ast }$ and ${Y}^{\ast }$ is included to allow for the association between *S* (and *E*) and ${Y}^{\ast }$to vary across levels of ${A}^{\ast }$and vice versa, reflecting the potential effect measure modification of the association between *A* and *Y* by *S* (and *E*). The regression coefficients of bias models are bias parameters.

2) **Impute the unobserved variables and derive selection weights for each individual**

The variables *U,A,Y* are singly imputed for each record based on the corresponding bias models in eqn (4) using the elicited values for the bias parameters and the observed data. In our example, binary *U,A,* and *Y* are imputed by sampling from a Bernoulli trial for each patient with success probability set to the predicted probability from the corresponding bias model.

Selection probabilities are estimated from the corresponding bias models for *E* and ${R}_y$ using the elicited values for the bias parameters and the observed data. These can be multiplied if assumed independent, as in Figure 3, and then used to derive inverse probability weights for each record in the analytic sample: $\hat{W}=1/\left[\hat{P}\left(S=1|{A}^{\ast },{Y}^{\ast}\right)\times \hat{P}\left(E=1|{A}^{\ast },{Y}^{\ast}\right)\times \hat{P}\left({R}_y=1|{A}^{\ast },{Y}^{\ast },\boldsymbol{C}\right)\right]$. These weights are used in the next step to create a pseudo-population that is representative of the target population.

3) **Perform weighted outcome regression**

The estimates of the causal risk ratio under the alternative assumptions can be obtained by fitting the outcome regression model in eqn (1) to the imputed data using weighting.

The uncertainty in the bias parameters can be incorporated in the final estimate using PBA. First, bias parameters are sampled from a plausible distribution over a number of replications and then Steps 2 and 3 are repeated for each draw, producing multiple bias-adjusted effect estimates. A simulation interval (SI) can be calculated using the range of estimates (e.g., the 2.5th and the 97.5th percentiles as the 95% SI).^3^ To also incorporate sampling variability into the SI, the whole procedure can be replicated over bootstrap samples or via a normal approximation as detailed elsewhere.^1^^,^^33^

#### Application to the case study

We used the above analytic method to analyze the multiple biases together and separately, i.e., one at a time, ignoring other biases. We used a probabilistic approach with 1000 draws. Where available, the key bias parameter distributions were obtained via a literature search and cross-checked with substantive context experts; otherwise, they were elicited from experts [see Table S2]. Other bias parameters were set to approximations based on the observed data. Sampling variability was incorporated by repeating the procedure over 1000 bootstraps.

Figure 4 shows results for the bias analyses alongside those for the primary analysis. The simultaneous multiple bias analysis resulted in somewhat stronger causal effect estimates, suggesting a larger reduction in risk of asthma under any breastfeeding vs not, compared with analyzing the biases separately, one at a time, or ignoring the potential biases, as in the primary analysis [also see Table S3]. Although the results across approaches were only minimally different in this example, this need not be the case in general as shown in the simulation study next.

**Figure 4:**
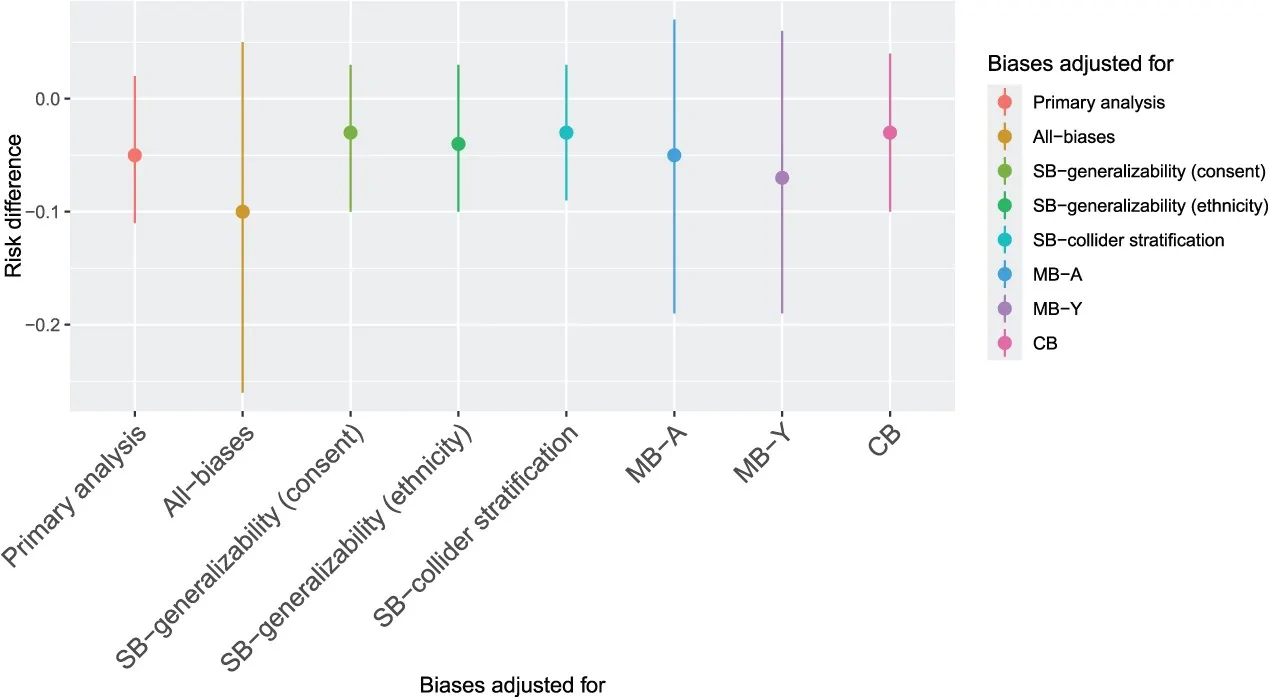
Point estimate and 95% confidence (in the primary analysis)/simulation (in all bias analyses) intervals from the application to the HealthNuts case study. Abbreviations: CB, confounding bias; MB, measurement bias; SB, selection bias.

### Simultaneous vs one-at-a-time analysis of multiple biases: simulation study

#### Simulation study aims and design

To demonstrate the need for analyzing multiple biases together, we conducted a proof-of-concept simulation study comparing 2 strategies for analyzing multiple biases: simultaneous and the more common one-at-a-time strategy.^35^

Data were generated and analyzed as detailed in Table 3, following a set-up similar to the case study with some simplifications (e.g., only one effect modifier leading to Type II SB). The key bias parameters are detailed in Table 4, with more details about the simulation design in Table S4.

**Table 3:** Proof-of-concept simulation study data design (see Table S1 for an extended table with additional details on the parameter values).

Simulated study	Details
Data generation	We simulated 1000 datasets (each of size *n* = 2000) using the parametric models detailed below based on Figure 3 in the main text. The non-bias model parameters were set to values estimated by fitting analogous models to the case study data (ignoring the unobserved variables). The values of the bias parameters (*pU,* $\mathrm{O}{\mathrm{R}}_{A-U},\mathrm{O}{\mathrm{R}}_{Y-U}, pE,\mathrm{O}{\mathrm{R}}_{\left(Y-A\right)E},\mathrm{O}{\mathrm{R}}_{Y-{R}_y},\mathrm{O}{\mathrm{R}}_{Y^{\ast }-Y},\mathrm{O}{\mathrm{R}}_{A^{\ast }-A},\mathrm{O}{\mathrm{R}}_{Y-{R}_y}$) were derived based on a literature review and cross-checking with substantive context experts (see Table S2 for relevant references). For simplicity, in the data generation under each scenario, we considered the bias parameters to be fixed. 1. Observed confounders $\boldsymbol{C}$were generated sequentially based on the hypothesized causal relationships structure in the published analysis. See Peters et al. ^6^ for more details. 2. Next, the unmeasured confounder, *U,* breastfeeding exposure *A*, Ability to speak/understand English *E,* and the outcome *Y* was generated using the following models, $U\sim \mathrm{Bernoulli}\left({\mathrm{logit}}^{-1}(pU)\right), A\sim \mathrm{Bernoulli}\left({\mathrm{logit}}^{-1}\left({\omega}_0+{\boldsymbol{\omega}}_{\mathbf{1}}^{\boldsymbol{T}}\boldsymbol{C}+\log \left(\mathrm{O}{\mathrm{R}}_{A-U}\right)\mathrm{U}\right)\right), E\sim \mathrm{Bernoulli}\left({\mathrm{logit}}^{-1}(pE)\right), Y\sim \mathrm{Bernoulli}\left({\mathrm{logit}}^{-1}\left({\mathrm{\phi}}_0+\log \left(\mathrm{\alpha} \right)A+{\mathrm{\phi}}_1^{\mathrm{T}}\boldsymbol{C}+\log \Big(\mathrm{O}{\mathrm{R}}_{Y-U}\right)U+0.18\mathrm{E}+\log \left(\mathrm{O}{\mathrm{R}}_{\left(Y-A\right)E}\right)A\times E\Big)\right),$where *α* was set to a fixed value determined via trial and error to achieve ~ 80% power for estimating the ACE using the target analysis models at the conventional 0.05 significance level in all scenarios (before introducing any missing values). Based on the above criterion, $\alpha =\left(-0.43\right)$ 3. Next, misclassified exposure and outcome were generated: ${A}^{\ast}\sim \mathrm{Bernoulli}\Big({\mathrm{logit}}^{-1}\left({\pi}_0+{\boldsymbol{\pi}}_{\mathbf{1}}^{\boldsymbol{T}}\boldsymbol{C}+\log \left(\mathrm{O}{\mathrm{R}}_{A^{\ast }-A}\right)A\Big)\right)$ ${Y}^{\ast}\sim \mathrm{Bernoulli}\Big({\mathrm{logit}}^{-1}\left({\lambda}_0+{\boldsymbol{\lambda}}_{\mathbf{1}}^{\boldsymbol{T}}\boldsymbol{C}+\log \left(\mathrm{O}{\mathrm{R}}_{Y^{\ast }-Y}\right)Y\Big)\right)$ 4. Finally, missingness in the outcome was generated: ${R}_Y\sim \mathrm{Bernoulli}\Big({\mathrm{logit}}^{-1}\left({\tau}_0+{\tau}_1A+{\tau}_2^TC+\log \left(\mathrm{O}{\mathrm{R}}_{Y-{R}_y}\right)Y\Big)\right)$
Estimand and methods	Following data generation, the one-at-a-time and simultaneous bias analysis strategies were applied to each dataset using the analytic approach described in section “Multiple Bias Modeling Approach” to estimate for the ACE of any breastfeeding on asthma at age 6 in the log risk ratio scale (i.e., $\log \left({\beta}_1\right)$)
Performance indicators	The “true” values of the target parameter in the log scale (i.e., ACE in log risk ratio scale) were obtained by fitting model eqn (1) to a very large sample without selection and all the unobserved data. These were used to compute the following performance measures: Bias*:* Average difference between the estimate from bias analysis and the true value Relative bias: bias relative to the true value as a percentage Empirical SE: SD of the estimate from the bias analysis Model-based SE: average of the estimated SE of the estimate from the bias analysis Coverage probability: proportion of replications where the estimated 95% CI contained the true value Bias-eliminated coverage: proportion of replications in which the estimated 95% CI contained the mean value of the estimate from bias analysis (across replications)

Abbreviations: ACE, average causal effect; CI, confidence interval; Emp SE, empirical standard error; OR, odds ratio.

Note: ${\mathrm{logit}}^{-1}\left(\left(\cdot \right)\right)=\exp \left(\cdot \right)/(1+\exp \left(\cdot \right)) $

**Table 4:** Bias parameter values used in the data generating models in the simulation study.

Bias	Parameter^a^	Parameter value
Selection bias (collider stratification bias)	Relative change in odds of asthma at age 6 in those whose guardian responded at age 6 vs not, $\mathrm{O}{\mathrm{R}}_{Y-{R}_y}$	1.20
Selection bias (Generalizability)	Probability of being able to speak and understand English $({p}_E)$ (marginal)	0.85
	Relative change in OR of asthma-breastfeeding in those who can speak/understand English compared to those who cannot, $\mathrm{O}{\mathrm{R}}_{\left(Y-A\right)E}$	0.70
Confounding bias due to unmeasured confounder gestational hypertension, *U*	Prevalence of gestational hypertension $({p}_U)$ (marginal)	0.10
	Relative change in odds of being ever breast-fed for infants with maternal gestational hypertension vs those without, $\mathrm{O}{\mathrm{R}}_{A-U}$	0.60
	Relative change in odds of having asthma at age 6 for infants with maternal gestational hypertension vs those without, $\mathrm{O}{\mathrm{R}}_{Y-U}$	1.30
Measurement bias exposure	Relative change in odds of parent reporting as ever breast fed in those ever-breast-fed vs never, $\mathrm{O}{\mathrm{R}}_{A^{\ast }-A}=\left[\mathrm{TP}/\mathrm{FN}\right]/\left[\mathrm{FP}/\mathrm{TN}\right]$ Where $\mathrm{TP}={p}_A\times N\times \mathrm{sens}$ $\mathrm{FN}=\left({p}_A\times N\right)-\mathrm{TP}$ $\mathrm{FP}=\left(1-{p}_A\right)\times N\times \left(1-\mathrm{spec}\right)$ $\mathrm{TN}=\left(\left(1-{p}_A\right)\times N\right)-\mathrm{FP}$ ${p}_A$ = 0.94	1.70 $\mathrm{sens}$= 0.90 $\mathrm{spec}$= 0.84
Measurement bias-outcome	Relative change in odds of parent reporting as child having asthma in those with asthma vs not, $\mathrm{O}{\mathrm{R}}_{Y^{\ast }-Y}=\left[\mathrm{TP}/\mathrm{FN}\right]/\left[\mathrm{FP}/\mathrm{TN}\right]$ Where TP, FN, FP and TN as above, calculated using ${p}_Y$= 0.10	0.54 $\mathrm{sens}$= 0.83 $\mathrm{spec}$= 0.90

Abbreviations: FN, false negatives; FP, false positives; OR, odds ratio; TN, true negatives; TP, true positives.

a Parameters are conditional unless stated otherwise and were often approximated using marginal values from the literature—see Table S2 for relevant references.

Performance of each strategy in terms of bias and precision were assessed using^36^: bias (average difference between the estimate and true value), relative bias (bias relative to the true value as a %), empirical standard error (SE) (SD of the estimate), model-based SE (average of the estimated SE of the estimate), coverage (proportion of 95% CI containing the true value), and bias-eliminated coverage (proportion of 95% CI containing the mean value of the estimate). Coverage summarizes both bias and variance estimation. That is, poor coverage could be due to bias and/or model-based SE*≠* empirical SE.^36^ Bias-eliminated coverage eliminates the bias of a method from CIs allowing to study CI coverage for mean of ${\hat{\beta}}_1$.

#### Simulation study results

As shown in Table 5, the simultaneous analysis of biases resulted in approximately unbiased estimates of the (log) risk ratio (<10% bias relative to the true value), while the primary analysis and all one-at-a-time bias-analyses produced estimates with comparatively large relative biases (>20%). This is expected of the primary analysis as it ignores all biases. It is also expected of the one-at-a-time approaches as these approaches considers each bias in isolation. For example, in assessing the bias due to unmeasured confounding, it is (implicitly) assumed that $A={A}^{\ast }$ and $Y={Y}^{\ast }$ and no selection in the sample has occurred based on *S,E,* and ${R}_y$*.* Thus, in generating values for *U*, the approach uses values of mismeasured exposure and outcome, ${A}^{\ast }$ and ${Y}^{\ast }$, within the observed portion of the study after selection. The simulations demonstrate that it is only by accounting for all of the sources of bias simultaneously that approximately unbiased estimates can be obtained.

**Table 5:** Comparative performance of the bias analysis strategies in estimating the log risk ratio, $\log \left({\beta}_1\right)$= log(0.51).

Bias analysis	Method	Average estimate	Bias	Relative bias	Emp SE	Model SE	Coverage	Bias eliminated coverage
	Primary analysis	−0.32	0.35	−52.76	0.50	0.66	0.96	0.93
One-at-a-time bias analysis	CB only	−0.31	0.35	−52.97	0.50	0.65	0.96	0.93
	MB-A only	−0.27	0.40	−59.66	0.24	0.23	0.63	0.94
	MB-Y only	2.60	3.27	−489.00	6.13	0.72	0.67	0.00
	SB-collider stratification only	−0.31	0.36	−53.45	0.50	0.65	0.96	0.94
	SB-generalizability only	−0.34	0.33	−49.71	0.51	0.66	0.97	0.93
Multiple bias analysis	All biases	−0.70	−0.04	5.30	0.36	0.33	0.92	0.94

Abbreviations: CB, confounding bias; Emp SE, empirical standard error; MB, measurement bias; Model SE; model-based standard error; SB, selection bias; SE, standard error.

Bias = average estimate-$\log \left({\beta}_1\right)$, relative bias = (bias/$\log \left({\beta}_1\right))\times 100\%,$ Emp SE = SD of the estimate, model SE = average of the estimated SE of the estimate, coverage = proportion of 95% CI containing the true value, bias-eliminated coverage = proportion of 95% CI containing the mean value of the estimate. All values apart from relative bias and empirical SE are mean values calculated from 1000 simulated datasets.

The simultaneous approach also resulted in appropriate nominal coverage (~ 0.93-0.96), which indicates proper variance estimation given the negligible bias. For most one-at-a-time approaches, some under- or overcoverage was observed, and these produced bias-eliminated coverage closer to nominal levels, indicating that the poor coverage observed was largely due to bias in point estimates.

## Discussion

In this paper, we introduced a 3-step roadmap for systematic identification and simultaneous analysis of multiple potential sources of bias, illustrated using a real case study. The steps include identifying all assumptions in the primary analysis through specification of the ideal trial and the assumptions required by its (possibly implicit) emulation,^17^ considering plausible assumption violations and depicting these in DAGs to identify the resulting causal biases where possible, and conducting a multiple bias analysis considering the biases simultaneously. Further, proof-of-concept simulations demonstrated how one-at-a-time bias analysis produce substantially biased causal estimates while simultaneous analysis yield approximately unbiased estimates with appropriate coverage (given correct bias model and parameters). The roadmap thus supports more robust and transparent QBA, particularly in settings where multiple potential sources of bias are present. Nevertheless, as with any bias analysis or indeed any causal inference, implementing the roadmap is not without challenges, as we discuss next.

The ideal trial in the first step of the roadmap is a hypothetical, perfect randomized experiment that would answer the ultimate scientific question of interest.^17^ As we discuss elsewhere,^17^ in specifying it, a balance must be struck between relevance to decision-making and feasibility of estimation with the available data. For instance, the outcome measure may be defined as the gold standard measure used in practice, even in the absence of this in the data. Focusing on the ideal trial is necessary for examining biases relative to the ultimate, practically relevant estimand.^17^ However, we recognize that striking this balance is not always straightforward. In our example, we considered these trade-offs when specifying the ideal trial.

In addition, in many settings, causal estimands that are meaningful and policy-relevant may be complicated by the presence of multiple versions of treatment. For example, in our case study, there are multiple versions of the “any breastfeeding” treatment strategy, corresponding to varying breastfeeding duration, frequency, and mode. Other examples include broad physical activity guidelines, where multiple versions arise due to type, intensity and frequency, or cancer screening guidelines with different screening modalities (e.g., for colorectal cancer screening, fecal immunochemical testing, stool DNA testing, or colonoscopy at varying intervals). As mentioned earlier, with such causal questions, where it can be assumed the treatment precedes version, identification remains possible under stronger assumptions than in settings with a single treatment version.^14^ Specifically, identification requires adequate control for factors that influence both the treatment and the treatment version.^14^ This assumption should be made explicit, and if violation is plausible, investigators could undertake further bias analyses to assess the potential impact. While such settings do not preclude the use of QBA or the proposed roadmap, they do introduce additional complexities that should be carefully considered.

Although DAGs play a prominent role in our roadmap and help clarify assumptions, they cannot identify (e.g., measurement bias) and/or demonstrate (e.g., Type II selection bias) all bias. Nevertheless, DAGs can help to clarify how the variables that play a role in biases arising are related. In some cases, researchers may have incomplete knowledge of the full data-generating mechanism, making it challenging to develop a DAG. Even in such situations, DAGs remain valuable for explicitly laying out assumptions, however uncertain, making the causal structure and alternative scenarios more transparent, and clarifying the potential pathways through which biases may arise.

Another obvious caveat in modeling multiple biases is the need to specify distributions for many bias parameters. Our case study demonstrates how investigators can reasonably source and justify these distributions [see Table S2], but further methodological work is needed, particularly for calibrating marginal parameters obtained from external sources to conditional bias parameters,^37^ to enable wider application. In addition, because bias parameters are often uncertain, treating them as fixed risks misspecification. Incorporating the uncertainty in bias parameters like we did via PBA is therefore recommended.^3^

All studies estimating causal effects either explicitly or implicitly make causal assumptions that, if violated, may result in biases with respect to the estimand of interest. Investigators should move beyond simply making qualitative statements about bias and quantitatively demonstrate the uncertainty of their results to multiple possible biases simultaneously. The roadmap presented offers a systematic guide to the necessary steps for achieving a more rigorous, comprehensive, and transparent bias analysis.

### Supplementary material

Supplementary material is available at the *American Journal of Epidemiology* online.

## Supplementary Material

Web_Material_kwag166

## Data Availability

All software code for the simulation study and case study analysis can be found here: [https://github.com/rushwije/MBA_roadmap]. Case study data cannot be made publicly available due to ethics requirements but are available from study investigators upon reasonable request. Please direct any case study data requests to health.nuts@mcri.edu.au.
