# Supplementary material for: Roadmap for systematic identification and analysis of multiple biases in causal inference
Source: Am J Epidemiol. 2026 Jul 16;195(11):2802–13. doi: 10.1093/aje/kwag166 (PMC13733541; doi:10.1093/aje/kwag166)
Supplement: Supplementary file 1 [file web_material_kwag166.zip › MBA_roadmap_supplementary.docx]

**Supplementary data file: Roadmap for systematic identification and analysis of multiple biases in causal inference**

**Authors:** Rushani Wijesuriya ^1,2*^, Rachael Hughes ^3,4^, John B Carlin^1,2^, Rachel L Peters^5.6^, Jennifer J Koplin ^6,7.^, and Margarita Moreno-Betancur ^1,2^

**ORCiD IDs:** Rushani Wijesuriya (0000-0003-1023-4065), Rachael Hughes (0000-0003-0766-1410), John Carlin (0000-0002-2694-9463), Rachel L Peters (0000-0002-2411-6628), Jennifer J Koplin (0000-0002-7576-5142), Margarita Moreno-Betancur (0000-0002-8818-3125)

Affiliations:

1. Clinical Epidemiology & Biostatistics Unit (CEBU), Murdoch Children's Research Institute
2. Clinical Epidemiology & Biostatistics Unit (CEBU), Department of Paediatrics, University of Melbourne
3. MRC Integrative Epidemiology Unit, University of Bristol
4. Population Health Sciences, Bristol Medical School, University of Bristol
5. Department of Paediatrics, University of Melbourne
6. Murdoch Children’s Research Institute
7. Child Health Research Centre, University of Queensland

*Correspondence: [r.wijesuriya@unimelb.edu.au](mailto:r.wijesuriya@unimelb.edu.au)

# Included material:

Table S1: Additional violations that were considered less plausible in the case study and justifications

Appendix S1 Bias-adjusting probability derivation

Table S2: Bias parameter distributions used in the case study application

Table S3: Application of the bias-adjustment strategies to the HealthNuts case study

Table S4: Simulation study design using the ADEMP structure [1]

## Table S1: Additional violations that were considered less plausible in the case study and justifications

| **Bias/violations** | **Justification** |
| --- | --- |
| Selection bias due to assuming that infants recruited via immunisation clinics to HealthNuts in 2007-2011 are representative of all infants aged 1 year born in Melbourne in 2006 -2010 | Given the high immunization rates in Victoria during this period^23^, and the low infant mortality^24^, we assume differences between the study sample and the target population due to these factors are minimal and resulting selection bias (if any) is negligible, |
| Positivity violation due to not excluding infants who may not be able to be breastfed, either due to infant being unable to ingest or digest breastmilk due to data not being available on this | We expect size of this group to be small (as it is a rare condition) and this the bias if any is expected to be small |
| Selection bias due to not excluding mothers unable to breastfeed, due to data not being available on this | We do not expect a different effect this group (i.e. no effect measure modification), so this bias is expected to be negligible |

## **Appendix S1** Bias-adjusting probability derivation

The desired joint probability refers to the joint probability of all variables that are required to perform the desired analysis while also encoding the alternative assumptions considered for the sensitivity analysis, i.e. $Y^{*}, A^{*},\boldsymbol{C}$, $Y,A, U$, as well as any selection variables that have been conditioned on in obtaining the observed data. The observed joint probability refers to the joint probability of the observed variables ($Y^{*}, A^{*},\boldsymbol{C)}$ in the selected study sample (i.e. in the stratum defined by $S=1, E=1 \mathrm{and} R_{y}=1)$

$$\text{bias-adjusting weight}=\frac{desired joint probability}{observed joint probability}$$

$$=\frac{P(Y=y,A=a,U=u{,A}^{*}=a^{*},Y^{*}=y^{*},\boldsymbol{C=c})}{P\left( Y^{*}=y^{*},A^{*}=a^{*},C=c \right|S=1,E=1,R_{y}=1)}$$

$$=\frac{P\left( Y=y,A=a,U=u{|A}^{*}=a^{*},Y^{*}=y^{*},\boldsymbol{C=c} \right)\times P(A^{*}=a^{*},Y^{*}=y^{*},\boldsymbol{C=c})}{P\left( Y^{*}=y^{*},A^{*}=a^{*},C=c \right|S=1,E=1,R_{y}=1)}$$

$$=\frac{P\left( Y=y,A=a,U=u{|A}^{*}=a^{*},Y^{*}=y^{*},\boldsymbol{C=c} \right)\times P(A^{*}=a^{*},Y^{*}=y^{*},\boldsymbol{C=c})}{\frac{P(S=1,E=1, R_{y}=1|A^{*}=a^{*},Y^{*}=y^{*},\boldsymbol{C=c})\times P(A^{*}=a^{*},Y^{*}=y^{*},\boldsymbol{C=c)}}{P(S=1,E=1, R_{y}=1)}}$$

$$\propto\frac{P(Y=y,A=a,U=u|A^{*}=a^{*},Y^{*}=y^{*},\boldsymbol{C=c})}{P(S=1,E=1, R_{y}=1|A^{*}=a^{*},Y^{*}=y^{*},\boldsymbol{C=c})}$$

$$= \frac{P(U=u|A=a,Y=y, A^{*}=a^{*},Y^{*}=y^{*},\boldsymbol{C=c})\times P(Y=y|A=a, A^{*}=a^{*},Y^{*}=y^{*},\boldsymbol{C=c})\times P(A=a|A^{*}=a^{*},Y^{*}=y^{*},\boldsymbol{C=c})}{P(S=1,E=1, R_{y}=1|A^{*}=a^{*},Y^{*}=y^{*},\boldsymbol{C=c})}$$

$$=\frac{P(U=u|A=a,Y=y)\times P(Y=y|A=a,Y^{*}\boldsymbol{=}y^{*}\boldsymbol{,C=c})\times P(A=a|A^{*}=a^{*},Y^{*}=y^{*},\boldsymbol{C=c})}{P(S=1,E=1, R_{y}=1|A^{*}=a^{*},Y^{*}=y^{*},\boldsymbol{C=c})}$$

… where the last step follows from the conditional independence assumptions encoded in Figure 3.

## Table S2: Bias parameter distributions used in the case study application

| **Bias** | **Bias parameter** | **Source(s)** | **Estimate** | **Conditional bias parameter distributions used in the case study** |
| --- | --- | --- | --- | --- |
| Confounding bias | Odds of gestational hypertension | Fox, Haylee, and Emily J. Callander. "The cost of hypertensive disorders of pregnancy to the Australian healthcare system." Pregnancy Hypertension 21 (2020): 197-199. <https://pubmed.ncbi.nlm.nih.gov/32634609/> | 0.03 | Normal distribution deduced by specifying 0.07 and 0.17 as the 2.5^th^ and 97.5^th^ percentiles and 0.12 as the mean[4]  $N(log(0.12),\frac{log(0.20)-log(0.07)}{2\times1.96})$ |
|  |  | Arnott, Clare, et al. "Maternal cardiovascular risk after hypertensive disorder of pregnancy." Heart 106.24 (2020): 1927-1933. <https://heart.bmj.com/content/106/24/1927> | 0.11 |  |
|  |  | Wertaschnigg, Dagmar, et al. "Hypertensive disorders in pregnancy–Trends over eight years: A population-based cohort study." *Pregnancy hypertension* 28 (2022): 60-65.  <https://www.sciencedirect.com/science/article/abs/pii/S2210778922000277> | 0.06 |  |
|  |  | Shaheen, Seif O., et al. "Hypertensive disorders of pregnancy, respiratory outcomes and atopy in childhood." European Respiratory Journal 47.1 (2016): 156-165.  <https://erj.ersjournals.com/content/erj/47/1/156.full.pdf> | 0.17 |  |
| Confounding bias | Odds ratio for the association between ever being breastfed and gestational hypertension | Horsley, Kristin, et al. "Hypertensive disorders of pregnancy and breastfeeding practices: A secondary analysis of data from the All Our Families Cohort." Acta Obstetricia et Gynecologica Scandinavica 101.8 (2022): 871-879.  <https://obgyn.onlinelibrary.wiley.com/doi/epdf/10.1111/aogs.14378> | 0.45 | $N\left( \log\left( 0.70 \right),\frac{\log\left( 0.80 \right)-\log\left( 0.50 \right)}{2\times1.96} \right)$ |
|  |  |  |  |  |
|  |  | Magnus, Maria C., et al. "Breastfeeding and later‐life cardiometabolic health in women with and without hypertensive disorders of pregnancy." Journal of the American Heart Association 12.5 (2023): e026696.  <https://www.ahajournals.org/doi/epub/10.1161/JAHA.122.026696> | 0.76 |  |
|  |  | Leeners B, Rath W, Kuse S, Neumaier-Wagner P. Breast-feeding in women with hypertensive disorders in pregnancy. J Perinat Med. 2005;33(6):553-60. doi: 10.1515/JPM.2005.099. PMID: 16318622.  <https://www.degruyter.com/document/doi/10.1515/JPM.2005.099/html> | 0.67 |  |
|  |  | Woolley, Emma, et al. "Exclusive Breastfeeding at Discharge in Regional New South Wales, Australia: The Role of Antenatal Care (2011–2020)." International Journal of Environmental Research and Public Health 20.12 (2023): 6135.  <https://www.mdpi.com/1660-4601/20/12/6135> | 0.60 |  |
| Confounding bias | Odds ratio for the association between asthma and gestational hypertension | Henderson, Ian, and Siobhan Quenby. "Gestational hypertension and childhood atopy: a Millennium Cohort Study analysis." European Journal of Pediatrics 180 (2021): 2419-2427.  <https://link.springer.com/article/10.1007/s00431-021-04012-3> | 1.25 | $N(\log\left( 1.1 \right),\frac{\log\left( 0.88 \right)-\log\left( 1.2 \right)}{2\times1.96})$ |
|  |  | Shaheen, Seif O., et al. "Hypertensive disorders of pregnancy, respiratory outcomes and atopy in childhood." European Respiratory Journal 47.1 (2016): 156-165.  <https://erj.ersjournals.com/content/erj/47/1/156.full.pdf> | 1.03 |  |
|  |  | Kelly, L., et al. "OP99 Hypertensive disorders in pregnancy and childhood diagnosis of asthma." (2019): A48-A49.  <https://jech.bmj.com/content/jech/73/Suppl_1/A48.2.full.pdf> | 1.37 |  |
|  |  | Ma, Ying, et al. "Associations between maternal complications during pregnancy and childhood asthma: a retrospective cohort study." ERJ Open Research 9.2 (2023).  <https://openres.ersjournals.com/content/erjor/9/2/00548-2022.full.pdf> | 1.65 |  |
| Measurement bias- breastfeeding self-reports | Sensitivity | Li, Ruowei, Kelley S. Scanlon, and Mary K. Serdula. "The validity and reliability of maternal recall of breastfeeding practice." Nutrition reviews 63.4 (2005): 103-110.  [The validity and reliability of maternal recall of breastfeeding practice - PubMed (nih.gov)](https://pubmed.ncbi.nlm.nih.gov/15869124/) | 0.82 | $beta(44,4)$  Beta distribution deduced by specifying 0.82 and 0.90 as the 2.5^th^ and 97.5^th^ percentiles and 0.84 as the prior mean[4] |
|  |  | Amissah, Emma Ayorkor, et al. "Validation study of maternal recall on breastfeeding duration 6 years after childbirth." Journal of Human Lactation 33.2 (2017): 390-400.  <https://www.ncbi.nlm.nih.gov/pmc/articles/PMC9353757/> | 0.91 (0.88-0.94) |  |
|  |  | Schneider, Bruna Celestino, et al. "Validation of maternal recall on exclusive breastfeeding 12 months after childbirth." Public health nutrition 23.14 (2020): 2494-2500.  [Validation of maternal recall on exclusive breastfeeding 12 months after childbirth - PMC (nih.gov)](https://www.ncbi.nlm.nih.gov/pmc/articles/PMC10200606/) | 0.98 (0.97-0.99) |  |
| Measurement bias- breastfeeding self-reports | Specificity | Li, Ruowei, Kelley S. Scanlon, and Mary K. Serdula. "The validity and reliability of maternal recall of breastfeeding practice." Nutrition reviews 63.4 (2005): 103-110.  [The validity and reliability of maternal recall of breastfeeding practice - PubMed (nih.gov)](https://pubmed.ncbi.nlm.nih.gov/15869124/) | 0.93 | $beta(82,18)$  Beta distribution deduced by specifying 0.70 and 0.85 as the 2.5^th^ and 97.5^th^ percentiles and 0.84 as the prior mean[4] |
|  |  | Amissah, Emma Ayorkor, et al. "Validation study of maternal recall on breastfeeding duration 6 years after childbirth." Journal of Human Lactation 33.2 (2017): 390-400.  <https://www.ncbi.nlm.nih.gov/pmc/articles/PMC9353757/> | 0.89 (0.84-0.92) |  |
|  |  | Schneider, Bruna Celestino, et al. "Validation of maternal recall on exclusive breastfeeding 12 months after childbirth." Public health nutrition 23.14 (2020): 2494-2500.  [Validation of maternal recall on exclusive breastfeeding 12 months after childbirth - PMC (nih.gov)](https://www.ncbi.nlm.nih.gov/pmc/articles/PMC10200606/) | 0.70 (0.68-0.72) |  |
| Measurement bias- breastfeeding self-reports | True population prevalence | Ogbo, Felix A., et al. "Prevalence and determinants of cessation of exclusive breastfeeding in the early postnatal period in Sydney, Australia." *International Breastfeeding Journal* 12 (2016): 1-10.  <https://link.springer.com/article/10.1186/s13006-017-0110-4> | 0.90 | $U(0.80,0.90)$  *note true population prevalence assumed to lower than values from literature based on self-reports |
|  |  | Netting, Merryn J., et al. "The Australian feeding infants and toddler study (OzFITS 2021): Breastfeeding and early feeding practices." *Nutrients* 14.1 (2022): 206.  <https://www.mdpi.com/2072-6643/14/1/206> | 0.93 |  |
|  |  | Scott, Jane, et al. "Determinants of continued breastfeeding at 12 and 24 months: results of an Australian cohort study." *International journal of environmental research and public health* 16.20 (2019): 3980.  <https://www.mdpi.com/1660-4601/16/20/3980> | 0.95 |  |
| Measurement bias- Child asthma parent-reports | Sensitivity | Hederos, Carl‐Axel, et al. "Comparison of clinically diagnosed asthma with parental assessment of children's asthma in a questionnaire." Pediatric allergy and immunology 18.2 (2007): 135-141. | 0.85 | $beta(54,13)$  Beta distribution deduced by specifying 0.77 and 0.90 as the 2.5^th^ and 97.5^th^ percentiles and 0.80 as the prior mean[4] |
|  |  | Jenkins, Mark A., et al. "Validation of questionnaire and bronchial hyperresponsiveness against respiratory physician assessment in the diagnosis of asthma." International journal of epidemiology 25.3 (1996): 609-616. | 0.77 |  |
|  |  | Valle, Solange Oliveira Rodrigues, et al. "Validity and reproducibility of the asthma core International Study of Asthma and Allergies in Childhood (ISAAC) written questionnaire obtained by telephone survey." Journal of Asthma 49.4 (2012): 390-394. | 0.87 (0.78-0.96) |  |
| Measurement bias- Child asthma parent-reports | Specificity | Hederos, Carl‐Axel, et al. "Comparison of clinically diagnosed asthma with parental assessment of children's asthma in a questionnaire." Pediatric allergy and immunology 18.2 (2007): 135-141. | 0.81 | $beta(42,5)$  Beta distribution deduced by specifying 0.81 and 0.95 as the 2.5^th^ and 97.5^th^ percentiles and 0.90 as the prior mean[4] |
|  |  | Jenkins, Mark A., et al. "Validation of questionnaire and bronchial hyperresponsiveness against respiratory physician assessment in the diagnosis of a sthma." International journal of epidemiology 25.3 (1996): 609-616. | 0.98 |  |
|  |  | Valle, Solange Oliveira Rodrigues, et al. "Validity and reproducibility of the asthma core International Study of Asthma and Allergies in Childhood (ISAAC) written questionnaire obtained by telephone survey." Journal of Asthma 49.4 (2012): 390-394. | 1.00 (0.90-1.00) |  |
| Measurement bias- Child asthma parent-reports | Population prevalence | Khan, J.R., Lingam, R., Owens, L. et al. Social deprivation and spatial clustering of childhood asthma in Australia. glob health res policy 9, 22 (2024). <https://doi.org/10.1186/s41256-024-00361-2> | 0.21 | $U(0.21,0.30)$ |
|  |  | Ahmad, K., Kabir, E., Ormsby, G.M. *et al.* Are wheezing, asthma and eczema in children associated with mother’s health during pregnancy? Evidence from an Australian birth cohort. *Arch Public Health* **79**, 193 (2021). <https://doi.org/10.1186/s13690-021-00718-w> | 0.26 |  |
|  |  | Thomson, Jennifer A., et al. "Early childhood infections and immunisation and the development of allergic disease in particular asthma in a high‐risk cohort: a prospective study of allergy‐prone children from birth to six years." *Pediatric allergy and immunology* 21.7 (2010): 1076-1085. <https://onlinelibrary.wiley.com/doi/full/10.1111/j.1399-3038.2010.01018.x> | 0.25(0.22-0.30) |  |
| Measurement bias- Child asthma parent-reports | Association between true asthma and true breastfeeding ^^^ | Lodge, Caroline J., et al. "Breastfeeding and asthma and allergies: a systematic review and meta‐analysis." Acta paediatrica 104 (2015): 38-53.  <https://doi.org/10.1111/apa.13132> |  | $N(\log\left( 0.6 \right),\frac{\log\left( 0.8 \right)-\log\left( 0.5 \right)}{2\times1.96})$ |
| Selection bias due to restricting study sample to those who consented | Odds of consent | Koplin, Jennifer J., et al. "Cohort Profile: The HealthNuts Study: Population prevalence and environmental/genetic predictors of food allergy." International journal of epidemiology 44.4 (2015): 1161-1171.  <https://doi.org/10.1093/ije/dyu261>  and expert opinion | 2.3-4 (prevalence 0.70-0.8) | $N(\log\left( 2.8 \right),\frac{\log\left( 4 \right)-\log\left( 2.3 \right)}{2\times1.96})$ |
|  | Odds ratio for the association between consent and breastfeeding | Elicited via expert opinion |  | $N(\log\left( 1.1 \right),\frac{\log\left( 1.3 \right)-\log\left( 1.0 \right)}{2\times1.96})$ |
|  | Odds ratio for the association between consent and asthma | Elicited via expert opinion |  | $N(\log\left( 1.2 \right),\frac{\log\left( 1.3 \right)-\log\left( 0.9 \right)}{2\times1.96})$ |
|  | Effect measure modification of consent -asthma by breastfeeding (Relative change in OR of consent -asthma in those who were ever breast-fed vs not) | Elicited via expert opinion |  | $N(\log\left( 1.4 \right),\frac{\log\left( 1.7 \right)-\log\left( 1.0 \right)}{2\times1.96})$ |
| Selection bias due to restricting study sample to those who read/understand English | Odds of ability to speak and understand English in Victoria from 2016-2021 | <https://profile.id.com.au/australia/speaks-english> | 4-9 (under prevalence 0.80-0.90) | $N(\log\left( 5 \right),\frac{\log\left( 9 \right)-\log\left( 4 \right)}{2\times1.96})$ |
|  | Odds ratio for the association between ability to read/understand English and breast feeding | Dennis, Cindy‐Lee, et al. "Breastfeeding rates in immigrant and non‐immigrant women: A systematic review and meta‐analysis." Maternal & Child Nutrition 15.3 (2019): e12809. <https://onlinelibrary.wiley.com/doi/full/10.1111/mcn.12809> | 0.88(0.84-0.93) | $N(\log\left( 0.88 \right),\frac{\log\left( 0.93 \right)-\log\left( 0.84 \right)}{2\times1.96})$ |
|  | Odds ratio for the association between ability to read/understand English and asthma | Wang, Yichao, et al. "Asian children living in Australia have a different profile of allergy and anaphylaxis than Australian‐born children: a State‐wide survey." *Clinical & Experimental Allergy* 48.10 (2018): 1317-1324.  <https://doi.org/10.1111/cea.13235> and expert opinion |  | $N(\log\left( 1.2 \right),\frac{\log\left( 1.5 \right)-\log\left( 0.90 \right)}{2\times1.96})$ |
|  | Effect measure modification of ability to read/understand English -asthma by breastfeeding (Relative change in OR of ability to speak English-asthma in those who were ever breast-fed vs not) | Elicited via expert opinion |  | $N(\log\left( 0.70 \right),\frac{\log\left( 0.80 \right)-\log\left( 0.5 \right)}{2\times1.96})$ |
| Selection bias due to missing data in the outcome | Odds of response for  parents/guardians with asthmatic children vs parents/guardians with non- asthmatic children | Elicited via expert opinion |  | $N(\log\left( 1.2 \right),\frac{\log\left( 1.5 \right)-\log\left( 1.10 \right)}{2\times1.96})$ |

* Conditional bias parameters were often approximated using marginal bias parameter values in the literature. Systematic approaches for calibrating marginal bias parameters to conditional bias parameters within this bias adjustment approach is still an area of further research.

^ Bias parameter only required for simultaneous bias adjustment

**Note:** Conditional bias parameter distributions for the observed confounder variables were estimated by fitting a logistic regression model for the corresponding observed variable (i.e. $Y^{*}$when imputing $Y$, and $A^{*}$ when imputing $A$) or the selection indicators with confounders as predictors

## Table S3: Application of the bias-adjustment strategies to the HealthNuts case study

|  |  | **Causal effect measure** | | | |
| --- | --- | --- | --- | --- | --- |
| **Approach** | **Biases adjusted for in bias analysis** | **Risk difference** | **95% SI** | **Risk ratio** | **95% SI** |
| Primary analysis | NA | -0.05 | -0.11, 0.02 * | 0.97 | 0.82,1.15 * |
| Simultaneous bias adjustment | All biases | -0.10 | -0.26, 0.05 | 0.77 | 0.54 ,1.14 |
| One-at-a-time bias adjustments | SB-collider stratification | -0.03 | -0.09, 0.03 | 0.82 | 0.56 ,1.37 |
|  | SB-generalizability (consent) | -0.03 | -0.1, 0.03 | 0.79 | 0.54,1.31 |
|  | SB-generalizability (English speaking/ethnicity) | -0.04 | -0.1,0.03 | 0.77 | 0.53 ,1.29 |
|  | MB-A | -0.05 | -0.19, 0.07 | 0.73 | 0.41 ,1.92 |
|  | MB-Y | -0.07 | -0.19, 0.06 | 0.86 | 0.64,1.14 |
|  | CB | -0.03 | -0.10, 0.04 | 0.81 | 0.57, 1.37 |

*95% CI shown

NA: Not applicable, CB: Confounding bias, MB: measurement bias, SB: Selection bias, SI: simulation interval

## Table S4: Simulation study design using the ADEMP structure [1]

|  | **Details**  *Recommendations from Table 1 of Morris et al. (Stat Med. 2019;38(11):2074-2102.), with additions* |
| --- | --- |
| **Aims** | 1. Compare the performance of the simultaneous and one-at-a-time bias-adjustment strategies |
| **Data generating mechanisms** | Data were simulated from parametric models, based on Figure 3 in the main text with model parameters estimated by fitting analogous models (ignoring the unobserved variables) as explained below Sample size (N): 2000 *Observed confounders* Observed confounders were generated sequentially as follows based on the hypothesized causal relationships structure in the published analysis. See [2] for more details : 1. First the following confounders were generated independently by randomly assigning a % of participants to categories based on observed proportions in each category in the real data. - family history of allergic disease $\left( \mathrm{FHx} \right):$ 31% No, 69% Yes - paternal asthma$\left( \mathrm{FPa} \right)$: 86% No,14% Yes - maternal asthma$\left( \mathrm{FMa} \right):$ 85% No,15% Yes - parental ethnicity$\left( \mathrm{PEth} \right):$ 60% Australian,10% Asian, 30% Other - number of older siblings$\left( \mathrm{NSibs} \right):$ 50% None, 33% One, 13% Two, 4% Three - child’s sex at birth$\left( \mathrm{Sex} \right):$ 51% Male,49% Female - SES$\left( \mathrm{SES} \right):$20% Quintile 1, 20% Quintile 2, 21% Quintile 3, 19% Quintile 4,20% Quintile 5 2. Maternal age at childbirth and maternal smoking were generated conditional on SES using a linear and logistic regression models as follows: $\mathrm{MAge}\sim N(30.82+2.20\mathrm{SE}S_{2}+2.14\mathrm{SE}S_{3}+2.65\mathrm{SE}S_{4}+3.75\mathrm{SE}S_{5},4.8)$; $\mathrm{MSmk}\sim Bernoulli({logit}^{-1}\left( -2.10-1.25\mathrm{SE}S_{2}-0.84\mathrm{SE}S_{3}-1.48\mathrm{SE}S_{4}-2.57\mathrm{SE}S_{5} \right))$ where $\mathrm{SE}S_{2}, SES_{3}, SES_{4},SES_{5}$ denotes indicator variables for $SES$ quintiles 2-5 3. Indicator for preterm birth was generated using a logistic regression model conditioning on maternal age at childbirth, SES, and maternal smoking: $\mathrm{GAge}\sim Bernoulli({logit}^{-1}\left( -2.51+0.01\mathrm{MAge}+0.04\mathrm{SE}S_{2}-0.16\mathrm{SE}S_{3}-0.31\mathrm{SE}S_{4}-0.16\mathrm{SE}S_{5}-0.08\mathrm{MSmk} \right))$ 4. Mode of delivery was generated using a logistic regression model conditioning on SES and maternal age at childbirth: $\mathrm{DMode}\sim Bernoulli({logit}^{-1}\left( -2.92+0.07\mathrm{MAge}-0.05\mathrm{SE}S_{2}-0.07\mathrm{SE}S_{3}-0.16\mathrm{SE}S_{4}-0.11\mathrm{SE}S_{5} \right))$ 5. Low birthweight was generated conditioning on mode of delivery, gestational age, SES, maternal smoking, maternal age at childbirth: $\mathrm{LBW}\sim Bernoulli({logit}^{-1}\left( -4.81+0.70\mathrm{DMode}+3.85\mathrm{GAge}+0.01\mathrm{MAge}+0.14\mathrm{SE}S_{2}-0.01\mathrm{SE}S_{3}+0.16\mathrm{SE}S_{4}+0.34SE+0.66\mathrm{MSmk} \right))$ Parameters in the models 1-5 above were closely based on associations observed in the real case study data [2] *Unmeasured confounder* 6. Gestational hypertension was generated independently from a binomial distribution using a success probability sourced from the literature (see Table 3 in main text) : $U\sim Bernoulli\left( p\_U \right)$ *Exposure (true unobserved)* 7. Breast feeding was generated using a logistic regression model conditioning on all observed confounders and unmeasured confounder as below: $A\sim Bernoulli({logit}^{-1}\left( 1.79-0.17Sex-0.56NSibs_{2}-0.99NSibs_{3}-1.06NSibs_{4}-0.38LBW+0.31PEth_{2}+0.36PEth_{3}+0.04FMa-0.11FPa-0.01FHx-0.10DMode+0.03GAge+0.03MAge+0.86\mathrm{SES}_{2}+1.08\mathrm{SES}_{3}+0.72\mathrm{SES}_{4}+0.81\mathrm{SES}_{5}-0.575MSmk+\log\left( OR_{A-U} \right)U \right))$ where $\mathrm{Et}h_{2},Eth_{3}$ denotes the indicator variables for Asian and other, while $\mathrm{Sib}s_{2}, Sibs_{3}, Sibs_{4}$ denotes indicator variables for two, three and four siblings Model parameters between exposure and observed confounders were based on associations observed in the real case study data using the mismeasured exposure and ignoring the unmeasured confounder, while associations between exposure and the unmeasured confounder ($OR_{A-U})$were set to values sourced from literature confirmed by substantive experts (see Table 3 in main text) *Selection- type II due to restricting analysis to those who can speak/understand English* 8. Ability to speak/understand English (yes/no) was generated from a binomial distribution using probability sourced from the literature (see Table 3 in main text) $E\sim Bernoulli\left( p\_E \right)$ *Outcome (true unobserved)* 9. Finally, the outcome was generated using a logistic regression model conditioning on all confounders (including the unmeasured confounder) and the true exposure (generated in step 7) and an interaction between exposure and ability to speak/understand English $Y \sim Bernoulli({logit}^{-1}\left( -2.39+\log\left( \alpha\right)A-0.63Sex+0.05Sibs_{2}+0.02Sibs_{3}-0.02Sibs_{4}+0.19LBW-0.04PEth_{2}+0.07PEth_{3}+0.95FMa+0.69FPa-0.11FHx+0.17DMode+0.19GAge+0.02MAge+0.11SES_{2}-0.05SES_{3}-0.12SES_{4}-0.02SES_{5}+0.80MSmk+log(OR_{Y-U} \right)U+0.18E+log(OR_{\left( Y-A \right)E}) A\times E)$ Model parameters between outcome and observed confounders were closely based on associations between observed in the real case study data using the mismeasured outcome and exposure, and ignoring the unmeasured confounder, missing data and effect modifier, while association between outcome and the unmeasured confounder ($OR_{Y-U})$ and regression coefficient for the interaction term were elicited via expert opinion (see Table 3 in main text). The regression coefficient for exposure log($\alpha)$was set to a fixed value determined via trial and error to achieve ~ 80% power for estimating the ACE using the target analysis model (1) in main text, at the conventional 0.05 significance level in all scenarios (before introducing any missing values). Based on the above criterion, under “realistic” scenario, $log(\alpha)=\left( -0.51 \right)$ *Misclassified exposure and outcome* 10. Misclassified exposure was generated using a logistic regression model conditional on the true exposure generated in step 7 and the observed confounders generated in steps 1-5. Model parameters between misclassified exposure and true exposure ($OR_{A^{*}-A})$were sourced from literature confirmed by substantive experts (see Table 3 in main text) and those between the misclassified exposure and observed confounders were closely based on associations in the case study. $A^{*} \sim Bernoulli({logit}^{-1}\left( 1.79-0.17Sex-0.56NSibs\_2-0.99NSibs\_3-1.06NSibs\_4-0.38LBW+0.31PEth\_2+0.36PEth\_3+0.04FMa-0.11FPa-0.01FHx-0.10DMode+0.03GAge+0.03MAge+0.86SES\_2+1.08+0.72SES\_4+0.81SES\_5-0.575MSmk+log(OR_{A^{*}-A} \right)A$) Where $OR_{A^{*}-A}=(TP/FN)/(FP/TN)$ and $\mathrm{TP}=p_{A}\times N\times sens,FN =(p_{A}\times N)-TP, \mathrm{TN}=\left( 1-p_{A} \right)\times N\times spec$ $, FP =\left( \left( 1-p_{A} \right)\times N \right)-TN$ $p_{A}=prevalence of A in the population=0.94$ $TP:true positives , TN:true negatives,FN:false negatives, FP: false positives,sens$: sensitivity, $spec:$ specificity. 11. Misclassified outcome was generated using a logistic regression model conditional on true outcome and the observed confounders with model parameters between the misclassified outcome and the true outcome ($OR_{Y^{*}-Y})$ sourced from literature confirmed by substantive experts (see Table 3 in main text) and those between the misclassified outcome and observed confounders based on associations in the case study. $Y^{*} \sim Bernoulli({logit}^{-1}\left( -2.39-0.63Sex+0.05Sibs\_2+0.02Sibs\_3-0.02Sibs\_4+0.19LBW-0.04PEth\_2+0.07PEth\_3+0.95FMa+0.69FPa-0.11FHx+0.17DMode+0.19GAge+0.02MAge+0.11SES\_2-0.05SES\_3-0.12SES\_4-0.02SES\_5+0.80MSmok+log(OR_{Y^{*}-Y} \right)Y$ Where $OR_{Y^{*}-Y}=(TP/FN)/(FP/TN)$ and $TP =p_{Y}\times N\times sens,FN =\left( p_{Y}\times N \right)-TP,TN =\left( 1-p_{Y} \right)\times N\times spec,FP =\left( \left( 1-p_{Y} \right)\times N \right)-TN$ $p_{Y}=prevalence of Y in the population=0.10$ $TP:true positives ,TN:true negatives,FN:false negatives,FP: false positives,sens: sensitivity, spec: specificity.$ *Selection- type I missingness in outcome* 12. Missingness in the outcome (i.e., an indicator variable for indicating missing or observed) was generated using a logistic regression model conditional on true exposure, true outcome and observed confounders. Model parameters for exposure and confounders were set to values close to those in observed data (using mismeasured exposure and ignoring outcome). The regression coefficient for the outcome, $OR_{Y-R_{y}},$were elicited via expert opinion. $R_{Y} \sim Bernoulli({logit}^{-1}\left( -1.74+0.79A-0.11Sex-0.19Sibs_{2}-0.30Sibs_{3}-0.52Sibs_{4}-0.18LBW-0.29PEth_{2}-0.35PEth_{3}+0.22FMa+0.21FPa+0.29FHx-0.13DMode+0.13GAge+0.06MAge+0.07SES_{2}+0.21SES_{3}+0.13SES_{4}-0.01SES_{5}-0.54MSmok+log(OR_{Y-R_{y}} \right)Y$ **Factors that were varied:** The strengths of the biases were varied by varying the magnitude of the following parameters: - Selection bias (collider stratification bias): $OR_{Y-R_{y}}$ - Selection bias (generalizability): $(p\_E$), $OR_{\left( Y-A \right)E}$ - Confounding bias: $p\_U$, $OR_{A-U}$, $OR_{Y-U}$ - Exposure misclassification bias: $OR_{A^{*}-A}$ - Outcome misclassification bias: $OR_{Y^{*}-Y}$ A "realistic value” was picked based on the literature and expert opinion (realistic scenario. See main text Table 3 for the values. |
| **Estimand/target of analysis** | - The estimand of interest is the ACE of breast-feeding at least once vs never on asthma (yes/no) at age 6 in the risk ratio scale - Each dataset was analysed using the model in (1), which are misspecified under the data generation. Thus, to avoid our results being affected by this misspecification bias, as “true” value for computing bias we used estimates for the exposure coefficient obtained by fitting these models to a very large sample. |
| **Methods** | For both (i) and (ii) below, the multiple bias adjustment method via imputation by Brendel et al [3] was used:  (i)one-at- time bias adjustment, producing multiple bias-adjusted estimates.  (ii)simultaneous adjustment of multiple biases producing a single bias-adjusted estimate |
| **Performance measures** | Bias: Average difference between the bias-adjusted estimate and the true value  Relative bias: Bias relative to the true value as a percentage  Empirical SE: Standard deviation of the bias adjusted estimate  Model-based SE: Average of the estimated standard error of the bias-adjusted estimate  Coverage probability: Proportion of replications where the estimated 95% confidence interval contained the true value  Bias-eliminated coverage: Proportion of replications in which the estimated 95% CI contained the mean value of the bias-adjusted estimate (across replications)  Note : Under-coverage could be due to bias and/or model-based SE$\neq$ empirical SE. Bias-eliminated coverage, eliminates the bias of a method from confidence intervals by studying confidence interval coverage for mean of $\hat{\beta}_{1}.$ [1] |

*${logit}^{-1}\left( \left( . \right) \right)=\exp\left( . \right)/(1+\exp\left( . \right))$

1.	Morris TP, White IR, Crowther MJ: **Using simulation studies to evaluate statistical methods**. *Statistics in medicine* 2019, **38**(11):2074-2102.

2.	Peters RL, Kay T, McWilliam VL, Lodge CJ, Ponsonby A-L, Dharmage SC, Lowe AJ, Koplin JJ: **The interplay between eczema and breastfeeding practices may hide breastfeeding's protective effect on childhood asthma**. *The Journal of Allergy and Clinical Immunology: In Practice* 2021, **9**(2):862-871. e865.

3.	Brendel P: **Applications of Multi-Bias Analysis in Studies of the Associations between Parkinson's Disease and Cancer**: University of California, Los Angeles; 2019.

4.	Fox MP, MacLehose RF, Lash TL: **Applying quantitative bias analysis to epidemiologic data**: Springer; 2021.
